# Effects of Angular Dependency of Particulate Light Scattering Intensity on Determination of Samples with Bimodal Size Distributions Using Dynamic Light Scattering Methods

**DOI:** 10.3390/nano8090708

**Published:** 2018-09-10

**Authors:** Haruhisa Kato, Ayako Nakamura, Shinichi Kinugasa

**Affiliations:** National Metrology Institute of Japan (NMIJ), National Institute of Advanced Industrial Science and Technology (AIST), Tsukuba Central 5, 1-1-1 Higashi, Tsukuba, Ibaraki 305-8565, Japan; a.nakamura@aist.go.jp (A.N.); s.kinugasa@aist.go.jp (S.K.)

**Keywords:** dynamic light scattering, size distribution, polystyrene latex, flow field-flow fractionation, bimodal

## Abstract

The angular dependency of light scattering intensity from differently sized particles strongly influences the apparent particle size distribution, as determined by dynamic light scattering (DLS) methods. Manufactured nanomaterials have size distributions more or less; therefore, the effect of detecting the angular dependency of the apparent size distribution by DLS is crucial. Commercial DLS instruments typically have two different types of detector angular position. The first is a detector angled at 90°, and the other is a backscattering angle detector. We therefore investigated the coverage and angular dependency when determining the relative concentrations of nanoparticles in polystyrene latex samples with a bimodal size distribution, using DLS methods both experimentally and theoretically. We used five differently sized polystyrene latex particles (one was a 70-nm nanoparticle and the others were various submicron-sized particles) in a variety of mixtures (the ratio of the difference of particle sizes ranged from approximately 2 to 7) to investigate the coverage and angular dependency of the recognition of the relative concentration ratio. In the case of size difference of approximately a factor of 2 or 3 between the two mixed particles (one was fixed at 70 nm), for DLS measurements at light scattering detector angles ranging from 60° to 150°, the homodyne photon correlation functions were approximately straight lines for mixtures of two differently sized polystyrene latex particles. The straight homodyne photon correlation functions were caused by the relatively strong light scattering from larger submicron particles masking the weaker light scattering from the smaller nanoparticles. As a result, DLS analysis could not recognize the relative concentration of nanoparticles in the mixture. In contrast to these samples, for mixtures of two differently sized polystyrene latex particles (one was 70 nm in size) with a size difference of a factor of 5, the homodyne correlation functions displayed an obvious curve for angles larger than 120°. This curve reflected an appropriate relative concentration ratio for the two differently sized polystyrene latex particles. Furthermore, for a mixture of two differently sized particles (one was again 70 nm) with size differences of a factor of 7, the homodyne correlation functions showed a clearly curved shape for detector angles larger than 90°, and yielded appropriate relative concentration ratios for the two different sizes of polystyrene latex particles. These observations were supported by theoretical investigation using Mie theory and asymmetric flow field-flow fractionation measurements with a multi-angle light scattering detector. Our investigation is crucial for achieving some degree of concordance on the determination of the size distribution of particles using DLS methods in industrial and academic fields.

## 1. Introduction

Dynamic light scattering (DLS) has been widely used in industrial and biological fields to determine the sizes of Brownian nano- and submicron-particles suspended in liquid phases [1,2,3,4,5,6,7,8,9,10,11]. The DLS method raises the possibility of in situ size determination of particles in liquids, and is fundamentally different from microscopic methods. In addition, DLS methods can yield measurements of ensemble-averaged particle sizes more quickly than microscopic methods. Because of the convenience and usability of DLS methods, many commercial instruments and analytical methods based on the various principles underlying DLS methods are available in nanotechnological and biological fields [12,13].

Although widely utilized in many fields, the reliability of the determination of size distributions of particles in liquid phases by DLS is not particularly good. This is largely because the measurement of the apparent diameters of materials over a wide size distribution using DLS methods usually depends on an analytical algorithm [11]. Furthermore, light scattered from large particles masks that from smaller particles, resulting in an overly large measured particle size when using DLS methods [12,14].

Manufactured materials have a wide size distribution; therefore, it is important to understand whether DLS methods supply an accurate size distribution of the particles or not. According to the previous investigation of the size distribution of submicron/micron-particles in liquid phases using DLS methods [14], it was found that DLS at an observed light scattering detector angle of 90° yields appropriate relative ratios of polystyrene latex particles of approximately 150 nm and 1000 nm suspended in a mixture. This suggests that, for such a range of particle sizes, DLS measured at an observed light scattering detector angle of 90° using an incident laser wavelength of 633 nm can stably and quantitively recognize the bimodal size distribution of a mixture of particles with size differences of approximately a factor of 6.

**Figure 1:**
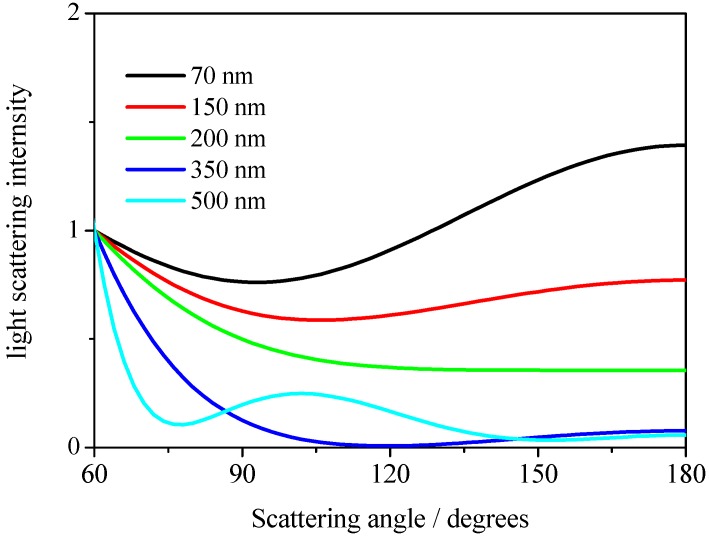
Light scattering intensity as a function of the scattering angle for different sizes of particle (70, 150, 200, 350, and 500 nm). The light scattering intensities are normalized by their theoretical values at a scattering angle of 60°. In this calculation, the refractive index of polystyrene latex particles was taken to be 1.59 and that of water was assumed to be 1.33.

However, the effect of detection angle on the apparent size distribution determined by DLS should be also considered, as commercial DLS instruments typically have two different detector positions when measuring light scattering. One is at 90° and the other is at a larger angle and measures backscattering. It is known that even for narrow size distributions of particles in liquid phase, the existence of electrostatic interactions between particles affects the angular distribution of particle scattering, resulting in an angular dependency of the apparent mean size measured by DLS [15,16]. In addition, a higher polydispersity induces larger differences in the observed particle size, depending on the light scattering detector angles, although this is also related to the relation between incident laser wavelength and targeted size in the DLS assessment. In Figure 1, the relative light scattering intensities as a function of the scattering angle are plotted using Mie theory [17]. In this calculation, refractive indices of 1.59 and 1.33 were used for polystyrene latex particle and water, respectively. In the figure, the light scattering intensity for each particle is normalized by its theoretical value at a scattering angle of 60°. As shown, the relative light scattering intensities at various angles are strongly dependent on particle size. This indicates that understanding the angular dependency of the apparent size distribution of particles in liquid phases in DLS assessment is crucial.

At present, for the regulation of many nanotechnological fields, the European Commission has declared that a “nanomaterial” is a natural, incidental, or manufactured material containing particles, in an unbound state or as an aggregate or agglomerate, in which 50% or more of the particles have one or more external dimensions in the size range 1–100 nm. According to this declaration, recognizing the capability of DLS methods to determine concentration ratios of nanoparticles in samples in manufacturing fields is significant. In this study, we examined the capabilities of DLS methods to determine the relative concentration ratios in nanoparticle samples with a bimodal size distribution using different light scattering detector angles, via both experimental and theoretical approaches. We prepared mixed particle suspensions containing two different sizes of polystyrene latex particles (one of which was a nanoparticle with a size of 70 nm) and qualitatively determined if the bimodal size distribution was observable using DLS methods at different light scattering detector angles. Quantitative discussion of the relative nanoparticle content of the mixtures allowed us to experimentally and theoretically examine the determination of the bimodal size distributions of mixtures using DLS. The recognition of the limitations and validity of DLS is essential and significant for both producers and users of materials. This quantitative estimation of the size distribution of particles using DLS will be of benefit to the fields of nano- and biotechnology.

## 2. Experimental Section

### 2.1. Materials

Aqueous suspensions of polystyrene latex particles with different mean sizes were purchased from Fujikura Kasei Co., Ltd., Kuki, Japan. The polystyrene latex particle suspensions used in this study were dispersed in surfactant-free aqueous solutions. The suspensions were diluted with ultrapure water prepared using the Puricω-system (Organo, Tokyo, Japan). The electric resistivity of the ultrapure water was 18.2 MΩ cm, and the total organic carbon contained in the water was less than 1 ppb. The characteristics of the suspension are summarized in Table 1. We also prepared mixtures of polystyrene latex particles and the size ratios of these mixtures for various particles are summarized in Table 2.

### 2.2. DLS Measurements and Analysis 

The DLS equipment (DLS8000HT, Otsuka Electronics Co., Ltd., Kyoto, Japan) used in this study had a goniometer equipped with a 45 mW He–Ne laser with a wavelength of 632.8 nm. In this study, DLS measurements were performed at light scattering detector angles of 60°, 90°, 120°, and 150°. A multiple-tau digital correlation scheme was used with a minimum sampling time of 0.1 µs. A quartz sample cell was set in a silicon oil bath such that the refractive indices of the oil and cell were almost equal. Light scattering was measured at a controlled temperature of 25.0 ± 0.1 °C. The values reported in this paper are expressed as the mean values of three consecutive measurements. The equipment was used in a clean booth maintained at a constant temperature of 23.0 ± 0.3 °C and humidity of 40% ± 3%.

### 2.3. Asymmetric Flow Field-Flow Fractionation with Multi-Angle Light Scattering Measurements

Asymmetric flow field-flow fractionation measurements (AF4) were carried out using an AF2000 FFF system (Postnova, Landsberg, Germany) equipped with a cellulose membrane (Z-MEM-AQU-427N, Postnova) with a 10,000 molecular mass cutoff filter and a channel thickness of 350 µm. The crossflow was controlled to perform the separation accurately, and the channel flow was held constant at a rate of 1.0 or 0.5 mL/min. The concentration of polystyrene latex particles in each fraction separated by AF4, based on the light scattering intensity at various scattering angles, was determined using a multi-angle light scattering (MALS) detector (Dawn HLEOS, Wyatt Co., Santa Barbara, CA, USA) at 690 nm. The MALS detector was calibrated using pure toluene, and the detections at different angles were normalized with respect to a 90° detector measuring a bovine serum albumin monomer separated by AF4.

## 3. Results and Discussion

### 3.1. DLS Measurements for Single Polystyrene Latex

**Figure 2:**
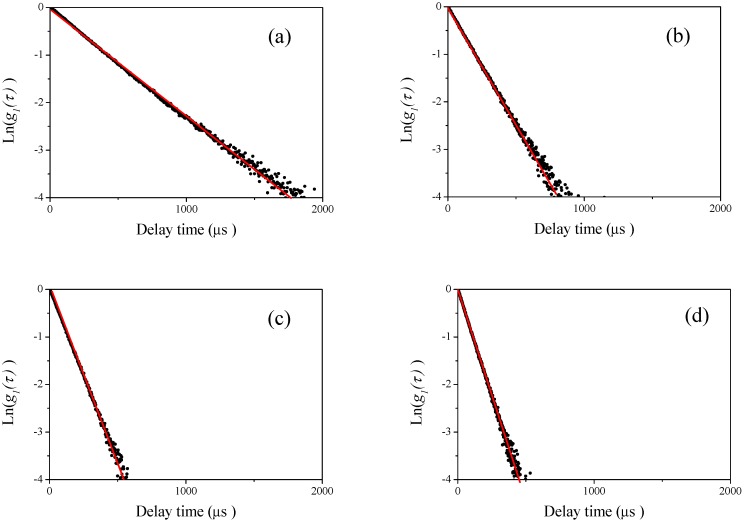
Examples of the observed homodyne photon correlation functions for polystyrene latex particles (W15BG001) using the dynamic light scattering (DLS) method (filled circles). The concentration of the aqueous polystyrene latex particle suspension was 0.1 mg/mL. The red line is the linear least-squares fit of the experimental results. (**a**) Detector angle of 60°; (**b**) detector angle of 90°; (**c**) detector angle of 120°; and (**d**) detector angle of 150°.

First, we performed DLS measurements at four different scattering angles of 60°, 90°, 120°, and 150° for single polystyrene latex samples. In Figure 1a–d, the example plots of the raw homodyne photon correlation functions of the corresponding polystyrene latex nanoparticle (W15BG001) obtained by DLS measurements (the concentration of the aqueous suspensions were 0.1 mg/mL) are shown. The filled circles in the figure indicate raw data, and the red line is a linear least squares fit with the observed photon correlation function for determining size using the well-known Stokes–Einstein assumption [12]. The raw photon correlation functions of the corresponding aqueous suspensions of polystyrene latex particles are straight lines for each observed scattering angle, indicating the narrow size distribution of the polystyrene latex samples. The calculated diameters of the polystyrene latex particles (W15BG001) were approximately 66.9 ± 0.8, 67.2 ± 0.6, 67.7 ± 0.5, and 68.4 ± 0.7 nm for scattering angles of 60°, 90°, 120°, and 150°, respectively. The error was calculated from three repeated DLS measurements. The difference in the values of the apparent size determined by DLS depending on the scattering angle were due to the existence of electrostatic interactions between particles [18,19] since the value of zeta potential of polystyrene latex is approximately −50 mV; slightly functionalized by COOH group. In DLS measurements for all the polystyrene latex particles, we found similar tendencies. For example, the calculated diameters of the polystyrene latex particles (W2KS150) were approximately 160.0 ± 1.2, 163.9 ± 1.4, 167.9 ± 1.0, and 170.7 ± 1.2 nm for scattering angles of 60°, 90°, 120°, 150°, respectively. The effects of polydispersity of the polystyrene latex particles and multiple scattering are not responsible for this dependency. The size distribution of the particles is not as large as that shown in Table 1, because such narrow polydispersity makes observed particle sizes larger at lower detecting angles in the Rayleigh region shown in Figure 2, and the sample did not exhibit multiple scattering effects at various incident beam intensities in this study. In addition, because the raw photon correlation functions of the polystyrene are straight lines, the observed tendency of the scattering angels are not caused by the polydispersity of the polystyrene latex particles. In the DLS measurements for the other three polystyrene latex samples, linear photon correlation functions were obtained for all samples at four detecting angles, indicating that all samples displayed a narrow size distribution assessed by DLS measurement.

**Figure 3:**
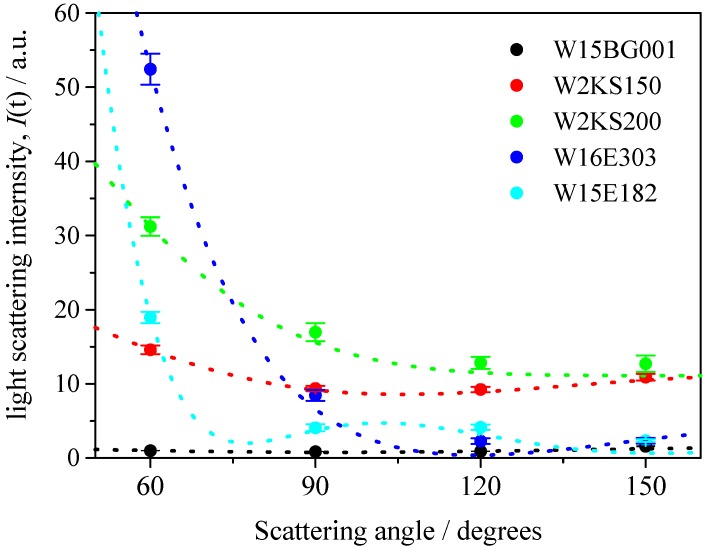
Plots of angler dependency of scattering intensity of polystyrene latex particles at the same mass (weight) concentration. The dotted lines are theoretical light scattering intensities for corresponding sized particles. The observed light scattering intensities were normalized by the intensity of W15BG001 at a scattering detection angle of 60°.

We then observed the dependency of the light scattering intensity on the observed detector angle for various polystyrene latex particles in aqueous media, as shown in Table 1. In Figure 3, the observed light scattering intensities are plotted at scattering angles of 60°, 90°, 120°, and 150°, respectively. The dotted lines are theoretical light scattering intensities from Mie theory [17] and Figure 1, and indicate that the observed intensities are in agreement with theoretical predictions (the observed light scattering intensities were normalized by the intensity of W15BG001 at a scattering detection angle of 60°). As shown in Figure 3, at smaller particle diameters such as 70 and 150 nm, the relative light scattering intensity at different detector angles obeyed the Rayleigh light scattering theory [20]. In contrast to these two particles, larger-sized polystyrene latex particles displayed obvious dependencies on the observed detector. This phenomenon was considered to be the origin of the angular dependency of DLS measurements for wider size distributions of particles.

### 3.2. DLS Measurements for Mixtures of Two Differently Sized Polystyrene Latex 

**Figure 4:**
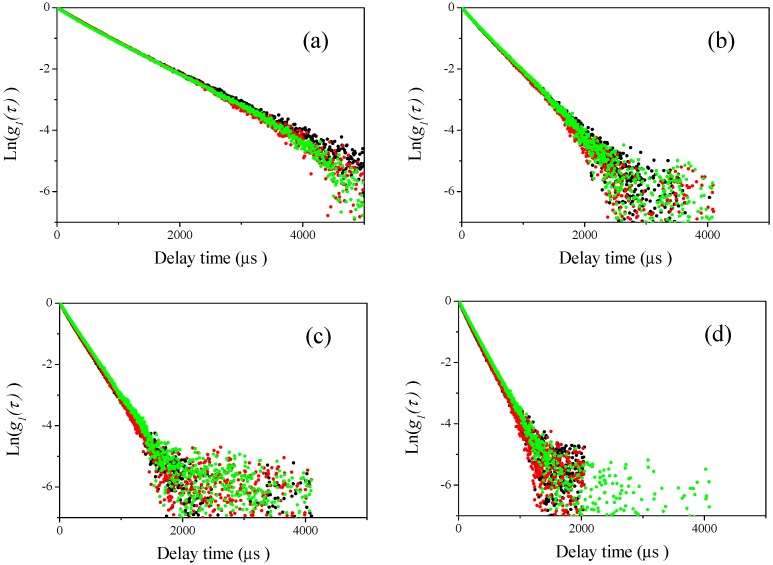
Examples of observed homodyne photon correlation functions for polystyrene latex particles measured by the DLS method (70-150-1 (black), 70-150-2 (red), and 70-150-3 (green)). (**a**) Light scattering was observed at a detector angle of 60°; (**b**) light scattering was observed at a detector angle of 90°; (**c**) light scattering was observed at a detector angle of 120°; and (**d**) light scattering was observed at a detector angle of 150°.

**Figure 5:**
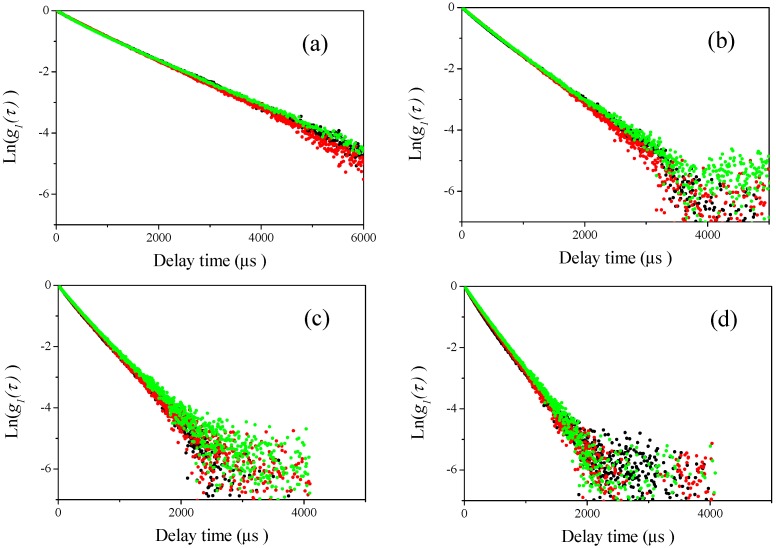
Examples of observed homodyne photon correlation functions for polystyrene latex particles by the DLS method (70-210-1 (black), 70-210-2 (red), and 70-210-3 (green)). (**a**) Light scattering was observed at a detector angle of 60°; (**b**) light scattering was observed at a detector angle of 90°; (**c**) light scattering was observed at a detector angle of 120°; and (**d**) light scattering was observed at a detector angle of 150°.

**Figure 6:**
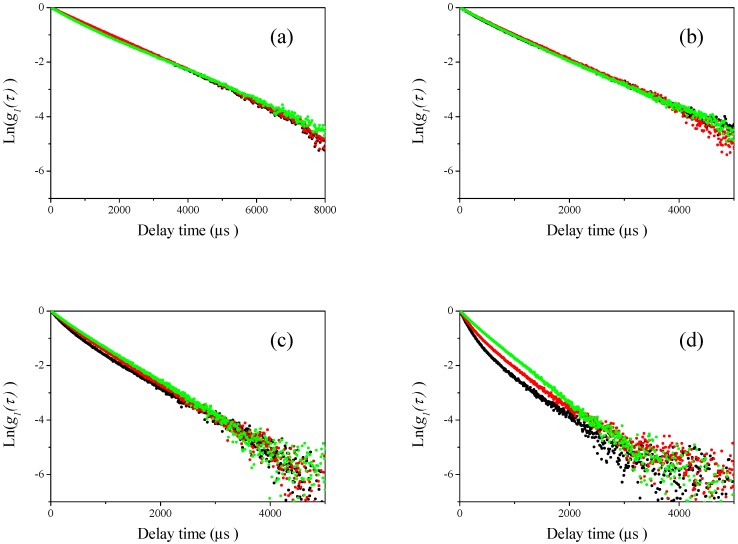
Examples of observed homodyne photon correlation functions for polystyrene latex particles measured with the DLS method (70-350-1 (black), 70-350-2 (red), and 70-350-3 (green)). (**a**) Light scattering was observed at a detector angle of 60°; (**b**) light scattering was observed at a detector angle of 90°; (**c**) light scattering was observed at a detector angle of 120°; and (**d**) light scattering was observed at a detector angle of 150°.

**Figure 7:**
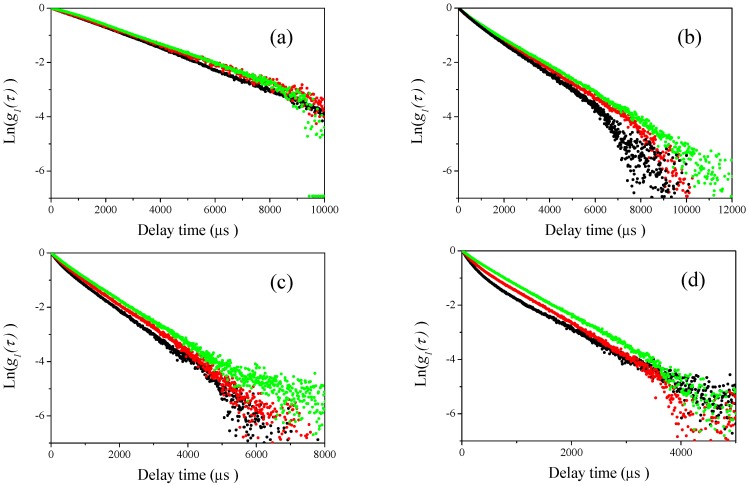
Example of observed homodyne photon correlation functions for polystyrene latex particles measured with DLS method (70-500-1 (black), 70-500-2 (red), and 70-500-3 (green)). (**a**) Light scattering was observed at a detector angle of 60°; (**b**) light scattering was observed at a detector angle of 90°; (**c**) light scattering was observed at a detector angle of 120°; and (**d**) light scattering was observed at a detector angle of 150°.

Then, DLS measurements of the sample mixtures were carried out at four different scattering angles (60°, 90°, 120°, and 150°, respectively) for the mixtures of different sizes of polystyrene latex particles shown in Table 2. The results are plotted in Figure 4, Figure 5, Figure 6 and Figure 7. In these figures, the black curves show the observed homodyne photon correlation functions for 2:1 polystyrene latex particles mixtures (70 nm and the respective submicron particle), the red colored curves are for 1:1 polystyrene latex particles mixture (70 nm and the respective submicron particle), and the green colored curves are for 1:2 polystyrene latex particles mixtures (70 nm and respective submicron sizes). Figure 4a, Figure 5a, Figure 6a and Figure 7a are the homodyne photon correlation functions observed at a light scattering detector angle of 60°. Figure 4b, Figure 5b, Figure 6b and Figure 7b are those for an angle of 90°. Figure 4c, Figure 5c, Figure 6c and Figure 7c are those for an angle of 120°. Figure 4d, Figure 5d, Figure 6d and Figure 7d are those for an angle of 150°. As shown in Figure 4 and Figure 5, the sample series 70-150 (70-150-1, 70-150-2, and 70-150-3) and 70-210 (70-210-1, 70-210-2, and 70-210-3) both display approximately straight homodyne photon correlation functions at all observed light scattering detector angles, indicating that it was difficult to recognize if the mixture of polystyrene latex particles had a monomodal or bimodal size distribution using the raw homodyne photon correlation functions when the size differences between the two mixed particles was a factor of approximately 2 or 3. The three homodyne photon correlation functions were similar in all figures, indicating that larger particles (150 and 210 nm) scattered more strongly than smaller nanoparticles (70 nm) and masked the smaller nanoparticles completely, resulting in all the homodyne photon correlation functions being overlapped. Indeed, the observed photon correlation functions were same as those from single larger submicron-sized particles. This observation indicates that the DLS method lacks the sensitivity to determine bimodal size distribution of particles in these size ranges.

In contrast to the two series of mixture samples 70-150 and 70-210, the results of the samples in the 70-350 series (70-350-1, 70-350-2, and 70-350-3) yielded substantially different observations, as shown in Figure 6. At the lower light scattering detector angles of 60° and 90° shown in Figure 6a,b, all the homodyne photon correlation functions overlapped as well as the 70-150 and 70-210 series shown in Figure 4 and Figure 5. However, at the larger detector angles of 120° and 150° shown in Figure 6c,d, obvious differences among three different mixture samples (70-350-1, 70-350-2, and 70-350-3) were observed. These results indicate that the raw homodyne photon correlation functions clearly distinguished the factor of 5 size differences between mixed particles at larger detector angles of 120° and 150°. Photon correlation curves, not linear structures, were observed, which are dependent on the ratio of the two different sizes of polystyrene latex particles. This indicates that the DLS method can distinguish the bimodal size distribution in mixture which has a size difference between particles of approximately a factor of five 5 at large detector angles of 120° and 150°.

Interestingly, as shown in Figure 7, the DLS measurement results of the samples in the 70-500 series (70-500-1, 70-500-2, and 70-500-3) demonstrated more obvious differences than those in the 70-350 series. At the lowest detector angle in this study (60°), the homodyne photon correlation functions approximately overlapped, though slight differences could be observed, as shown in Figure 7a. However, at three larger detector angles (90°, 120°, and 150°), obvious differences between three different mixture samples (70-500-1, 70-500-2, and 70-500-3) were observed. Raw homodyne photon correlation functions reflecting the factor of seven size difference between particles were clearly distinguished at detector angles of 90°, 120°, and 150°. At these angles, photon correlation curves were observed, not linear structures, and there was no visible overlap between the three photon correlation functions which were observed. These curves depended on the relative concentration of the two different sizes of polystyrene latex particles, similar to those in Figure 6c,d. These results indicate that DLS is not sufficiently sensitive to distinguish size differences from raw homodyne photon correlation functions when the particles in a mixture have a size difference less than a factor of 3. However, DLS easily distinguished particles with size differences greater than a factor of 5 at large detector angles for samples containing two different sizes of polystyrene latex particles. As the relative light scattering intensities of the differently sized particles at larger detector angles are smaller than at lower detector angles, as shown in Figure 3, the masking effect of light scattering predominantly from larger submicron particles is reduced. This means that the raw homodyne photon correlation functions displays the size differences between mixed two particles more readily than at lower detector angles. Commercial DLS instruments typically have two different detectors for measuring the angular position of light scattering: one is at 90° and the other is in the backscattering position. Our results indicates that the backscattering detector in DLS instruments is better-suited to recognize the existing of nanoparticles in particle suspensions with bimodal size distributions.

### 3.3. Determination of The Relative Concentration Ratios of Nanoparticles in Samples

To perform theoretical curve-fitting of the raw homodyne photon correlation curves for the 70-350 and 70-500 samples, we simply considered a reconstruction model of two different photon correlation functions as described by Equations (1) and (2). Namely, by assuming the sum of two different photon correlation functions between noninteracting particles.

(1) $$g_{1}(\tau )=xg_{1}{\left(\tau \right)}_{A}+\left(1-x\right)g_{1}{\left(\tau \right)}_{B}$$

(2) $$\frac{I{\left(t\right)}_{A}}{I{\left(t\right)}_{A}+I{\left(t\right)}_{B}}=x$$

In these equations, $g_{1}\left(\tau \right)$ is the homodyne photon correlation functions, *I*(*t*) is the light scattering intensities, and the subscripts represent the ID of the particles. In this assessment, the series 70-150 and 70-210 were not considered as the observed photon correlation functions did not reflected scattering from 70-nm-sized particles, as shown in Figure 4 and Figure 5. In this study, we already observed the relative light scattering intensities of polystyrene latex particles in our light scattering system as shown in Figure 3; therefore, by using just these two simple equations, the determination of the relative ratios of the mass concentration of nanoparticles could be obtained.

**Figure 8:**
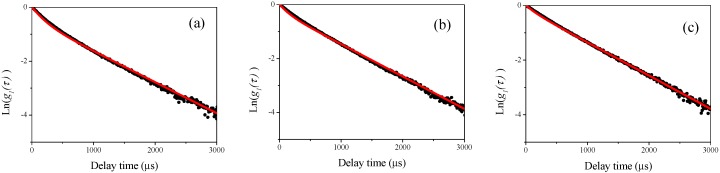

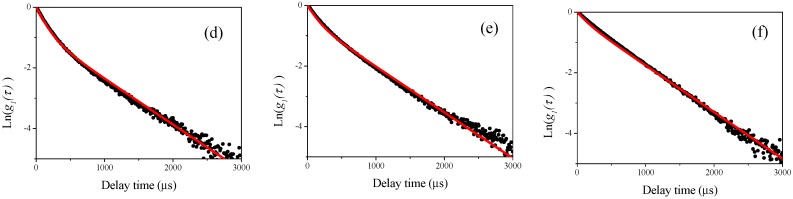
Observed homodyne photon correlation functions (black dots) vs. calculated homodyne photon correlation function curve using equations 1 and 2 (red solid curves) for the results shown in Figure 6c,d. (**a**) 70-350-1 at a detector angle of 120°; (**b**) 70-350-2 at a detector angle of 120°; (**c**) 70-350-3 at a detector angle of 120°; (**d**) 70-350-1 at a detector angle of 150°; (**e**) 70-350-2 at a detector angle of 150°; and (**f**) 70-350-3 at a detector angle of 150°.

**Figure 9:**
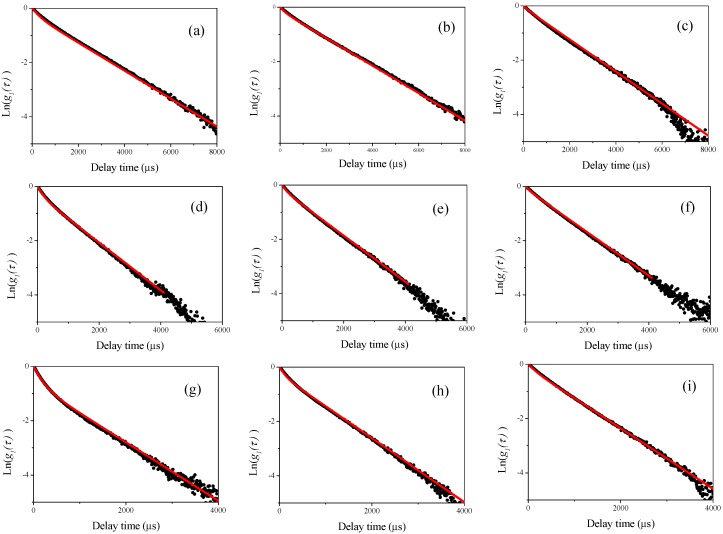
Observed homodyne photon correlation functions (black dots) vs. calculated homodyne photon correlation function curve using equation 1 and 2 (red solid curves) for the results shown in Figure 6c,d. (**a**) 70-500-1 at a detector angle of 90°; (**b**) 70-500-2 at a detector angle of 90°; (**c**) 70-500-3 at a detector angle of 90°; (**d**) 70-500-1 at a detector angle of 120°; (**e**) 70-500-2 at a detector angle of 120°; (**f**) 70-500-3 at a detector angle of 120°; (**g**) 70-500-1 at a detector angle of 150°; (**h**) 70-500-2 at a detector angle of 150°; and (**i**) 70-500-3 at a detector angle of 150°.

In Figure 8 and Figure 9, we show the curve-fitting performed using Equations (1) and (2) on the observed homodyne photon correlation functions. The black curves are the observed homodyne photon correlation functions from Figure 6 and Figure 7. Using Equations (1) and (2) and the $g_{1}\left(\tau \right)$ obtained from the homodyne photon correlation functions for the single particle samples listed in Table 1, the theoretical homodyne photon correlation functions were calculated, as shown by the red solid curves. The curve-fitting could not be applied to the results of Figure 6a,b and Figure 7a, as obvious differences between the photon correlation functions were not found due to the effect of light scattered from larger submicron-sized particles masking the light scattered from 70-nm-sized polystyrene latex nanoparticles.

Interestingly, by changing the values of *x* in the curve fitting Equation (2), the determined relative concentration ratios of W15BG001, using the relationship between light scattering intensity and mass concentration of particles shown in Figure 3, gave an appropriate relative concentration ratio. In this assessment, because it was difficult to analytically determine the uncertainty of the curve fitting procedure using Equations (1) and (2), we changed the values of *x* and compared the fitted theoretical curves and the experimental homodyne photon correlation functions. We then evaluated the maximum and minimum values of *x* that agreed with the observed homodyne photon correlation functions as shown in Figure 8 and Figure 9. The calculated uncertainties are described in Table 3. The calculated relative concentration ratios of W15BG001 in each mixture were quite appropriate, namely 67% for 70-350-1 and 70-500-1, 50% for 70-350-2 and 70-500-2, and 33% for 70-350-3 and 70-500-3, respectively. The experimental homodyne photon correlation function curves agreed well with the calculated homodyne photon correlation curves, which reflect the ratios of directly observed light scattering intensities from the two differently sized particles with two different photon correlation functions under the simple assumption of approximately noninteracting photon correlation functions. As shown in Table 3, the calculated uncertainties became larger using the observed photon correlation functions at lower detecting angles. These results again indicate that the backscattering detector position in DLS equipment has higher sensitivity than lower detection angles when determining the relative concentrations of nanoparticles in a sample of polystyrene latex particles with a bimodal size distribution.

### 3.4. Asymmetric Flow Field-Flow Fractionation Measurements for Mixtures of Two Differently Sized Polystyrene Latex

**Figure 10:**
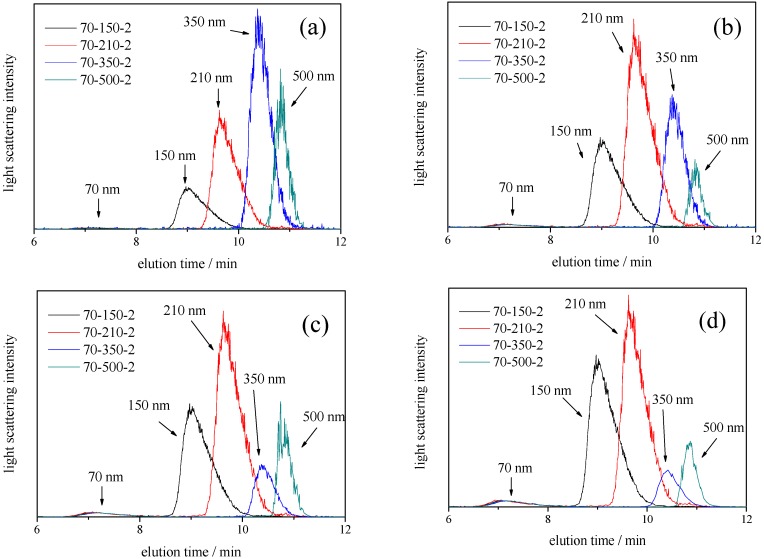
Fractograms observed at for mixed polystyrene latex particle suspensions by (fractionation measurements) AF4 separation. The channel flow rate was 1.0 mL/min and a cross-flow gradient of 2.0 mL/min to 0.0 mL/min over 30 min was used. 70-150-1 (black), 70-210-2 (red), 70-350-2 (blue), and 70-500-2 (green). Light scattering intensities were obtained by MALS. (**a**) observed at a detector angle of 60°; (**b**) observed at a detector angle of 90°; (**c**) observed at a detector angle of 121.2°; and (**d**) observed at a detector angle of 152.5°.

Finally, to recognize the phenomena observed by DLS in this study, we performed AF4-MALS measurements for four samples (70-150-2, 70-210-2, 70-350-2, and 70-500-2). AF4 is an elution technique in which particles are separated by flow control in an aqueous solution [21,22,23]. In AF4, the retention time *t_r_* of nanoparticles can be predicted by Giddings’s theory [21], according to which the retention time of a nanoparticle is given by,

(3) $$t_{r}=\frac{\pi \eta dw^{2}}{2kT}\frac{V_{C}}{V_{0}}$$

where *kT* is the thermal energy, *η* is the carrier elution viscosity, *d* is the diameter of the nanoparticle, *w* is the channel thickness, *V*_0_ is the volumetric flow rate of the channel flow, and *V_C_* is the cross-flow rate. The result is that the retention time is related to the size of the particles. In this study, we selected four different detection angles (60°, 90°, 120°, and 150°) for DLS measurements; therefore, we plotted the AF4-MALS fractograms for four samples at similar detection angles (60°, 90°, 121.2°, and 152.5°) as shown in Figure 10. As the detection angles were already fixed in the MALS instrument system, we selected similar angles in the Figure 10 as detector angles in Figure 4, Figure 5, Figure 6 and Figure 7. Because the AF4 is a separation system, the fractograms from a light scattering detector using four different angles would give the relative light scattering intensities from each differently sized polystyrene latex particles without the masking effect by larger particles observed in the DLS measurements in Figure 4, Figure 5, Figure 6 and Figure 7. This means that the observed fractograms are directly comparable with the results of DLS at similar scattering angles. The results from AF4-MALS must therefore provide valuable information to validate the ability of DLS measurement to determine bimodal size distributions of particles, including nanoparticles and submicron particles. Interestingly, after size fractionation by AF4 for four mixture samples, all samples clearly indicated the presence of two differently sized particles (both nanoparticle and submicron particles) without the masking effect observed in DLS measurements. Furthermore, it was clearly observed that (1) the relative light scattering intensities of larger submicron-sized particles (150, 210, 350, and 500 nm) to 70 nm nanoparticles became smaller with increasing detection and that (2) the relative light scattering intensities of larger particles (150, 210, 350, and 500 nm) changed depending on their sizes as well as the results shown in Figure 3. These results support the results of DLS measurements presented in this study, and indicate that the appropriate selection of the detector angles is important when determining the relative concentration ratio of nanoparticles in the samples with a bimodal size distribution. In addition, as the relative light scattering intensities for differently sized particles were strongly depending on the detector angle, not only DLS but also AF4-MALS assessment should take into account such effects, though it is a well-known result of Mie theory [17], as shown in Figure 1.

## 4. Conclusions

The determination of the relative concentrations of nanoparticles in samples with a bimodal size distribution of particles using DLS methods was investigated both experimentally and theoretically. As commercial DLS instruments typically have two different detector positions (one at 90° and the other measuring backscattering at large angles), it is important to recognize the angler dependency of sensitivity when performing DLS assessment of nanomaterials. In this study, an obvious angular dependency was observed in the homodyne photon correlation functions of mixtures of particles. DLS equipment could not recognize the bimodal size distribution of the mixture of two different sizes of polystyrene latex at any detection angle when the size difference between particles was a factor of 2 or 3. However, the recognition of bimodal size distribution was observed for mixtures of two differently sized particles when the size differences between mixed two particles was a factor of 5, though only for detection angles larger than 120°. For mixtures of two differently sized particles with size differences of a factor of 7, a bimodal size distribution could be detected by DLS at detection angles larger than 90°. These results indicate that positioning detectors at a large angle is advantageous when using DLS to determine the relative concentration of nanoparticles in the samples with bimodal size distributions when compared to lower detection. The theoretical investigation using Mie theory and experimental results from AF4-MALS assessment clearly showed that the relative light scattering intensities of larger particles to smaller nanoparticles became smaller with increasing detector angle, supporting the results from DLS measurements in this study.

## Figures and Tables

**Table 1:** Characteristics of polystyrene latex particles used in this study.

Sample Name	Official Diameter ^(a)^	Official CV Value ^(b)^
	(nm)	%
W15BG001	70	6.1
W2KS150	156	6.3
W2KS200	210	4.7
W16E303	350	5.1
W15E182	500	5.0

^(a)^ The official values of diameter were determined by dynamic light scattering (DLS); ^(b)^ The official Coefficient of Variation (CV) values were calculated from the standard deviation of the size distribution by SEM, and the observed size distributions were divided by size.

**Table 2:** Characteristics of polystyrene latex particle mixtures used in this study.

Sample Name	Sample	Sample
	Relative Ratio of W15BG001	Relative Ratio of W2KS150
70-150-1	2	1
70-150-2	1	1
70-150-3	1	2
70-210-1	2	1
70-210-2	1	1
70-210-3	1	2
70-350-1	2	1
70-350-2	1	1
70-350-3	1	2
70-500-1	2	1
70-500-2	1	1
70-500-3	1	2

**Table 3:** Determined relative concentration ratio (wt.%) of W15BG001 in a mixture of two polyethylene latex particles.

Sample Name	Observed Angle (°)			
	60	90	120	150
70-350-1	−	−	67 ± 6	67 ± 3
70-350-2	−	−	50 ± 5	50 ± 3
70-350-3	−	−	33 ± 6	33 ± 3
70-500-1	−	67 ± 10	67 ± 5	67 ± 2
70-500-2	−	50 ± 9	50 ± 5	50 ± 2
70-500-3	−	33 ± 9	33 ± 6	33 ± 3
